# Warthin-Like Papillary Carcinoma of the Thyroid Gland: Case Report and Review of the Literature

**DOI:** 10.1155/2012/689291

**Published:** 2012-12-02

**Authors:** Panagiotis Paliogiannis, Federico Attene, Federica Trogu, Mario Trignano

**Affiliations:** Department of Surgical, Microsurgical and Medical Sciences, University of Sassari, 07100 Sassari, Italy

## Abstract

We present a case of Warthin-like papillary thyroid carcinoma in a 22-year-old woman and a review of the literature on the topic. The patient had the occasional discovery of a hypoechoic thyroid nodule of approximately 18 mm, characterized by irregular margins, hyperechoic spots, rich intra- and perilesional vascularization, and a suspicious enlarged right laterocervical lymph node. Fine-needle aspiration was performed for both lesions and the diagnosis of papillary thyroid carcinoma without lymph node involvement was made. The patient underwent thyroidectomy and central neck lymphadenectomy without complications. Histopathological examination suggested a Warthin-like papillary carcinoma of the thyroid gland, with all the removed lymph nodes being free of disease. The patient subsequently underwent iodine ablative therapy and she remains free of disease one year after surgery. Warthin-like papillary thyroid carcinoma is a recently described variant of papillary thyroid cancer that is frequently associated with lymphocytic thyroiditis. Morphologically, it resembles Warthin tumors of the salivary glands, with T and B lymphocytes infiltrating the stalks of papillae lined with oncocytic cells. Surgical and postoperative management is identical to that of classic differentiated thyroid cancer, while prognosis seems to be favourable.

## 1. Introduction

We present a case of a Warthin-like papillary thyroid tumor (WaLPTT) in a 22-year-old woman with a family history of thyroid goitre. WaLPTT is a rare variant of papillary thyroid carcinoma (PTC) with approximately 80 cases reported in literature to date [1]. It was first described in 1995 by Apel et al., who noticed in their series of 13 cases morphological resemblance to Warthin's tumour of the salivary glands [2]. Through our case we describe the clinical and diagnostic features and surgical and oncologic management of patients with WaLPTT and present a review of the literature.

## 2. Case Presentation

In December 2010, a 22-year-old woman underwent an ultrasound (US) examination of the thyroid as a practical demonstration in the medical school of the local university where she studies medicine. The examination showed a gland that was normal in size with a heterogeneous parenchymal echostructure. In the inferior pole of the right lobe, a hypoechoic nodule of approximately 18 mm in greatest diameter was detected. It was characterized by irregular margins, hyperechoic spots and rich intra- and perilesional vascularization. Several enlarged lymph nodes were also found bilaterally in the laterocervical regions, but only one node on the right side was suspicious for metastasis on contrast US.

The patient had a family history of thyroid goitre, but no previous exposure of the neck to radiation or other risk factors for thyroid cancer. Physical examination did not reveal any significant alteration of the thyroid gland. No cervical lymph node swelling was detected by palpation, despite the ultrasonographic findings. Routine laboratory tests revealed slight hypothyroidism (FT4: 0.66 ng/dL, range 0.77–2.19) and a raised anti-TPO antibody level (519.0 UI/mL, range 0.00–10.00), suggesting chronic autoimmune thyroiditis.

A computed tomography (CT) scan of the neck and mediastinum confirmed the presence of several lymph nodes of approximately 10 mm in size bilaterally in the laterocervical and submandibular regions, most of which were oval in shape. No lymph node enlargement in the mediastinum or pulmonary or bone metastatic lesions was observed.

Fine-needle aspiration (FNA) showed numerous multinucleated and micropapillary cell groups, with voluminous nuclei and often with pseudoinclusions and a small number of lymphocytes. The diagnosis was PTC. Neoplastic involvement of the suspicious right laterocervical lymph node was excluded through FNA.

**Figure 1:**
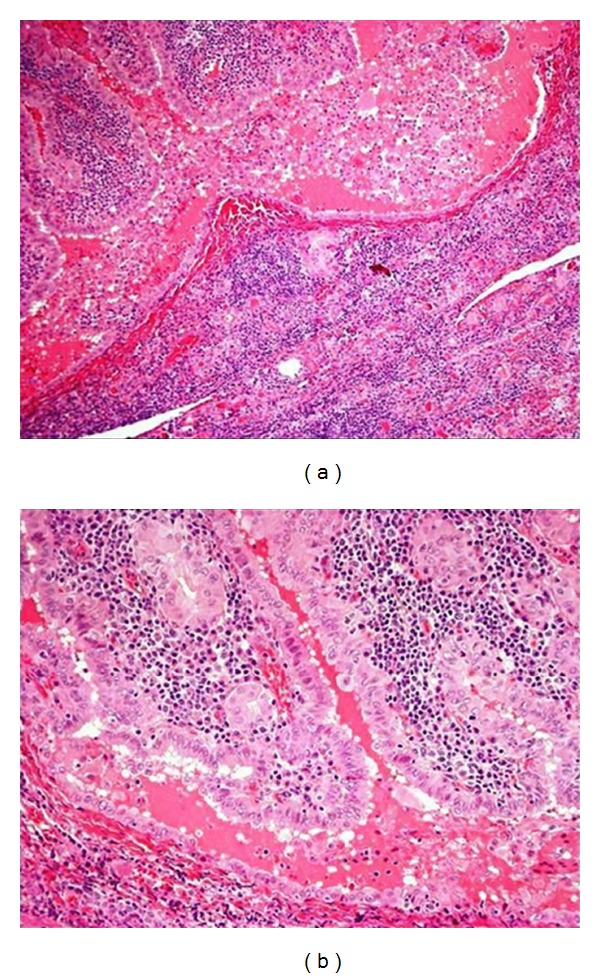
Hematoxilin and Eosin, stained sections of the tumor.

The patient underwent total thyroidectomy on March 2011. Central neck lymphadenectomy was performed, as at least two swollen nodes were detected in that area. The nodes were sent for frozen section pathological examination, which showed the absence of tumoral invasion. Pathological analysis of the specimen confirmed the presence of an 18 mm moderately differentiated papillary carcinoma with abundant Warthin-like lymphoid infiltration and capsular invasion. Furthermore, the gland was affected by chronic lymphocytic thyroiditis (Figure 1). Immunohistochemistry was not performed as the morphological picture was clear.

There were no postoperative complications and the patient was discharged on the second postoperative day. She underwent I^131^ radio-ablative therapy (106 mCi), as scintigraphy performed three months after surgery showed residual areas of activity in the neck, while her plasmatic thyroglobulin level was 12.5 ng/mL. One year after surgery, the blood thyroglobulin level was almost undetectable and the patient was stable and disease-free.

## 3. Discussion

The Warthin-like variant of papillary thyroid carcinoma was first described in 1995 by Apel et al. [2] and represents a rare variant of PTC, with approximately eighty cases having been reported in literature to date [1]. Apel et al. chose the name “Warthin-like tumor” because of its histological resemblance to papillary cystoadenoma lymphomatosum or Warthin tumor of the salivary glands.

Patients affected by WaLPTT have similar demographic and clinical characteristics to those affected by PTC.  Table 1 shows these characteristics, as reported in the most consistent series published to date, excluding case reports [2–7]. WaLPTT typically arises at an earlier age, as compared to PTC, and there is also a stronger predominance of females. Among the 54 patients reviewed, the mean age was 50 years and there were only five males (9%). The clinical presentation is the same as that for other differentiated thyroid tumors: absence of signs and symptoms when the lesions are single, small, and deep; palpable masses, glandular swelling, and swallowing and/or phonatory alterations for larger, superficial, and/or multiple lesions. Signs, symptoms, and alterations in thyroid function related to thyroiditis or goitre may be also present. Furthermore, features of US and CT imaging of WaLPTT are identical to those for PTC. As a consequence, diagnosis of WaLPTT cannot be based on clinical and imaging data alone.

FNA may be useful in this regard, but its role in the diagnosis of WaLPTT is not yet clear. Generally, it is possible to appreciate the presence of groups of follicular cells and papillary fragments against a background of lymphocytes and plasma cells, which infiltrate the fibrovascular cores. Oncocytic cells are often admixed with lymphocytes. The nuclear characteristics of tumoral cells are generally those of PTC (nuclear chromatin clearing, membrane thickening, grooves, and pseudoinclusions) and oncocytes (round nuclei with coarse chromatin and prominent nucleoli) [5]. Such patterns suggest PTC or lymphocytic thyroiditis, or both. Indeed, these were the most frequent diagnoses in the literature on patients affected by WaLPTT. Baloch and LiVolsi report data on seven FNAs; there were four diagnoses of PTC, two of lymphocytic thyroiditis and one of atypical cells [5]. In the series of D'Antonio et al., all patients underwent FNA and two of them had a diagnosis of PTC; in one case, the results of FNA were inconclusive [4]. In our case and those of Amico et al., Sarkady et al., and Lam et al., the preoperative diagnosis based on FNA was PTC or thyroiditis [1, 8, 9]. FNA is probably more useful for the evaluation of cervical lymph node involvement, as in our case, and for planning the appropriate surgical strategy and multidisciplinary management.

The macroscopic appearance of WaLPTT is generally that of a white-greyish, well circumscribed nodule, unencapsulated and confined to the thyroid gland. It may contain cystic and/or haemorrhagic areas. Its mean size among the 54 reviewed patients was 1.5 cm (range 0.3 to 5 cm). To our knowledge, only one paper has reported a WaLPTT larger then 5 cm [1]. The color of the remaining thyroid parenchyma generally ranges from red brownish to tan and a variable number of nodules of different sizes may be present.

The histological diagnosis of WaLPTT is based on the evidence of a morphological pattern featuring papillae lined by oncocytic cells admixed with lymphocytes and sparse plasma cells. The distinctive feature is the lymphocytic infiltration of the stalks of the papillae. Furthermore, it is possible to observe cysts or a small number of tall cells. Vascular and capsular invasions are rare. Differential diagnosis must be conducted with other variants of papillary cancer with similar morphology, such as Hurthle cell carcinoma (HCC) and tall cell carcinoma (TCC). The former usually lacks lymphoplasmacytic infiltrates and is rarely associated with lymphocytic thyroiditis [2, 5]; the latter is characterized by a papillary structure with elongated oncocytes, with a height that is more than twice their width, and by neoplastic aggressiveness with more frequent vascular, capsular, and nodal invasion [10].

The role of immunohistochemistry in differential diagnosis with Hurthle cell and tall cell carcinomas is limited. Indeed, in our case it was not necessary for diagnosis as the morphological pattern was clear. Intense staining for the following markers has been reported in literature: galectin-3, HBME-1, CK19, TTF-1, thyroglobulin, EMA, AE1/AE3, S-100 protein, cyclin D1 and UCHL1, CD3+, CD20+, and CD79+ (for the lymphocytic population). Ostrowski and Merino believe that CD15 immunostaining represents a distinctive characteristic of tall cell carcinomas and a predictive factor for advanced stage or poor prognosis [11]. Nevertheless, this marker was also found in WaLPTT, which has a better prognosis than TCC.

The neoplastic behaviour of  WaLPTT seems to be similar or even better than that of classical PTC. Vera-Sempere et al. hypothesized that it is a hybrid of TCC and HCC and some immunohistochemical evidence supports this theory [12]. Short- and long-term prognosis seems to be excellent. Lam et al. describe a case of a 74-year-old Chinese woman affected by WaLPTT with dedifferentiated (anaplastic) area [9]. She had a 3.5 cm PTC of the right lobe with ipsilateral recurrent laryngeal nerve involvement; a supraclavicular lymph node was shown by FNA to be metastatic. The patient refused any treatment for three years, but agreed to undergo surgery when her condition worsened. She underwent palliative surgery and died 15 months later. This case seems to have involved the natural evolution of an advanced anaplastic thyroid carcinoma (with areas of WaLPTT) that remained untreated for a long time, rather than a typical WaLPTT. Elsewhere, Amico et al. reported a case of a 6 cm WaLPTT with less than 5% of the tumor area being occupied by anaplastic tissue in a 79-year-old woman with laterocervical lymph node metastasis [1]. The patient promptly underwent surgery and was alive without evidence of disease 23 months later. It is probable that in such situations the anaplastic component, even when it represents a small percentage of the overall tumor area, dictates the prognosis, which may be poor when appropriate treatment is not provided.

Other cases of cervical nodal metastasis have been reported in literature, but such events are generally rare [3, 8]. Among the 54 cases reviewed here, only 12 (22%) had lymph node metastasis, which represents an incidence lower than that for traditional PTC. Prognosis was favourable in almost all cases, but follow-up times were generally short, with only a few cases being followed for more than 3 years. The most reliable explanation for the low rates of nodal involvement and favourable prognosis in WaLPTT is the presence of lymphatic tissue within the tumor, which seems to contrast and restrain neoplastic progression and diffusion.

The therapeutic management of patients with WaLPTT is similar to that of patients with PTC and depends substantially on the disease stage and the presence of negative prognostic factors such as familiarity, history of neck irradiation, and syndromic endocrine disease. The diagnosis of WaLPTT is histopathological and generally based on surgical specimens. This means that the therapeutic plan for patients with WaLPTT must be assessed after surgery. The reports reviewed in this paper are mostly pathological and lack detailed data on postoperative treatments such as radioiodine ablation and follow-up modalities. As the biological behaviour of WaLPTT is comparable to that of PTC, postoperative management should also be identical. Further surgery for completion of thyroidectomy or lymphadenectomy, radioiodine ablation, and other treatments may be employed in high-risk patients. Our patient underwent postoperative radioiodine therapy due to evidence of residual thyroid tissue and capsular invasion and is undergoing the same postoperative follow-up and treatment program used in patients with classic PTC.

## 4. Conclusions

Warthin-like papillary thyroid carcinoma is a recently described variant of papillary thyroid cancer that is frequently associated with lymphocytic thyroiditis. Morphologically, it resembles Warthin tumors of the salivary glands, with T and B lymphocytes infiltrating the stalks of papillae lined with oncocytic cells. Diagnosis is histopathological and based on morphology rather than immunohistochemistry. Surgical and postoperative management is identical to that of classic differentiated thyroid cancer, while prognosis seems to be favourable. Larger prospective long-term studies are necessary to better understand the biological behaviour of such tumors and their clinical and prognostic impact.

## Figures and Tables

**Table 1:** Demographic and clinical data of patients with WaLPTT as depicted in the literature.

Report	No. of cases	Mean age (range)	Sex (M-F)	Mean size (range)	Lymphocytic thyroiditis	Lymph node involvement
Apel et al. (1995) [2]	13	44 years (26–66)	1-12	1.6 cm (0.3–3.5)	10 (77%)	3 (23%)
Tazawa et al. (1999) [3]	4	50 years (49–53)	0-4	1.9 cm (1.5–3)	4 (100%)	4 (100%)
D'Antonio et al. (2000) [4]	3	50 years (43–56)	1-2	1.4 cm (1.3–1.5)	3 (100%)	0 (0%)
Baloch et al. (2000) [5]	17	42 years (23–63)	2-15	1.3 cm (0.3–3.5)	17 (100%)	3 (18%)
Ludvíková et al. (2001) [6]	12	64 years (45–85)	1-11	2.7 cm (1–5)	11 (92%)	2 (17%)
Kim et al. (2006) [7]	5	52 years (33–65)	0-5	1.5 cm (0.9–2)	4 (80%)	0 (0%)

Total	54	50 years (23–85)	5-49	1.5 cm (0.3–5)	49 (91%)	12 (22%)
